# The effect of concha bullosa and nasal septal deviation on palatal dimensions: a cone beam computed tomography study

**DOI:** 10.1186/s12903-021-01974-6

**Published:** 2021-11-23

**Authors:** Shishir Ram Shetty, Saad Wahby Al Bayatti, Natheer Hashim Al-Rawi, Vinayak Kamath, Sesha Reddy, Sangeetha Narasimhan, Sausan Al Kawas, Medhini Madi, Sonika Achalli, Supriya Bhat

**Affiliations:** 1 Department of Oral and Craniofacial Health Sciences, College of Dental Medicine, University of Sharjah, Sharjah, United Arab Emirates; 2 Goa Dental College and Hospital, Bambolim, Goa India; 3 College of Dentistry, Gulf Medical University, Ajman, United Arab Emirates; 4 Manipal College of Dental Sciences, Manipal Academy of Higher Education, Manipal, India; 5 A B Shetty Memorial Institute of Dental Sciences, Nitte Deemed to be University, Mangalore, India

**Keywords:** Nasal septum, Concha bullosa, Palate, Turbinate, Cone beam computed tomography

## Abstract

**Introduction:**

Nasal septal deviation (NSD) and concha bullosa (CB) are associated with airway obstruction in mouth breathers. Mouth breathing is associated with alterations in maxillary growth and palatal architecture. The aim of our study was to determine the effect of the presence of CB and NSD on the dimensions of the hard palate using cone-beam computed tomography (CBCT).

**Materials and methods:**

A retrospective study was conducted using CBCT scans of 200 study subjects. The study subjects were divided into four groups based on the presence of CB and NSD. Septal deviation angle (SDA), palatal interalveolar length (PIL), palatal depth (PD) and maxillopalatal arch angle (MPAA) were measured in the study groups.

**Results:**

The presence of NSD and CB was associated with significant (*p* < 0.001) differences in the palatal dimensions of the study subjects. The PIL and MPA (*p* < 0.001) were significantly reduced (*p* < 0.001), whereas the PD was significantly increased (*p* < 0.001) in study subjects with NSD and CB. There was no significant change in the palatal dimensions between the unilateral and bilateral types of CB. Among the palatal dimensions, the PIL had the most significant association (R^2^ = 0.53) with SDA and CB. There was a significant correlation between the palatal dimensions and SDA when CB was present along with NSD.

**Conclusion:**

Based on the results of this study, it can be concluded that the presence of NSD and CB have a significant effect on the palatal dimensions and, therefore, they may be associated with skeletal malocclusion.

## Introduction

Mouth breathing secondary to nasal passage obstruction has a major effect on the formation of dento-facial structures [1, 2]. Although enlarged adenoids are the primary cause of mouth breathing, nasal septal deviation (NSD), concha bullosa (CB) and inferior turbinate hypertrophy (TH) have also been implicated as other mechanical obstruction factors [3]. A significant association between CB and contralateral NSD has been reported by recent studies [4]. NSD is thought to enhance the pneumatization of the middle turbinate depending on the degree of deviation angle [5]. Nasal obstruction caused by NSD induces nasal airflow turbulence that leads to nasal dryness and recurrent sinusitis and turbinate thickening (TH) [6]. Studies have indicated that an insufficient nasal airway can lead to persistent mouth breathing during the developmental years, causing varying degrees of maxillary constriction [7]. Mouth breathers show a narrower hard palate than nasal breathers [8]. However, there is a scarcity of radiographic studies evaluating the palatal dimensions of subjects with CB, TH and NSD. The evaluation of the palatal dimensions and nasal structures is important from a clinical aspect, as procedures such as rapid maxillary expansion (RME) have a significant effect on nasal structures [9]. With this background, we conducted a study to determine the effect of CB and NSD on the dimensions of the hard palate using CBCT.

## Materials and methods

A retrospective evaluation of 200 CBCT scans of subjects who had attended University Dental Hospital Sharjah (UDHS) clinics for various dental treatments from January 2018 to December 2020 was carried out. Ethical approval for the study was obtained from the institutional ethical committee of the University of Sharjah (Reference number: REC-21-01-10-01). The CBCT scans of the male and female study subjects between 18 and 75 years of age were included in this study.

The CBCT scans were obtained using a Galileos Comfort CBCT unit (Sirona Dental Systems, Bensheim, Germany). The imaging parameters were field of view (17 × 13 cms), 85 kVp, 7 mA, 14 s exposure and voxel size 0.25 mm. The CBCT scans were viewed using the SIDEXIS Operating system on a 1920 × 1080 pixel and 23-inch DELL monitor screen.

Two dental radiologists with 10 years of experience examined the CBCT scans. A third examiner with equivalent expertise was consulted in cases of a disagreement between the two primary examiners. The scans were re-examined by the same radiologists after a duration of one month to determine the intraobserver reliability.

CBCT scans of unacceptable image quality were not included in this study. Three CBCT scans with partial images and six CBCT scans with streak artefacts were also excluded. CBCT scans of patients with a history of maxillofacial trauma (n = 1), sinonasal tumours (n = 1) and cleft palate (n = 1) were excluded from this study.

The included CBCT scans were categorized into four groups based on the presence of CB and NSD:

- Group 1: (n = 90) CBCT scans of subjects with no SD and CB.
- Group 2: (n = 55) CBCT scans of subjects with NSD only.
- Group 3: (n = 32) CBCT scans of subjects with CB only.
- Group 4: (n = 23) CBCT scans of subjects with NSD and CB.

Sample size estimation (n = 200) was performed using statistical Software G*Power 3.1. Based on observations made in the previous literature [Kajan et al., 2016] and considering the effect size, power and α error of 5%, a minimum sample size of 20 was considered appropriate for the subgroups.

**Fig. 1:**
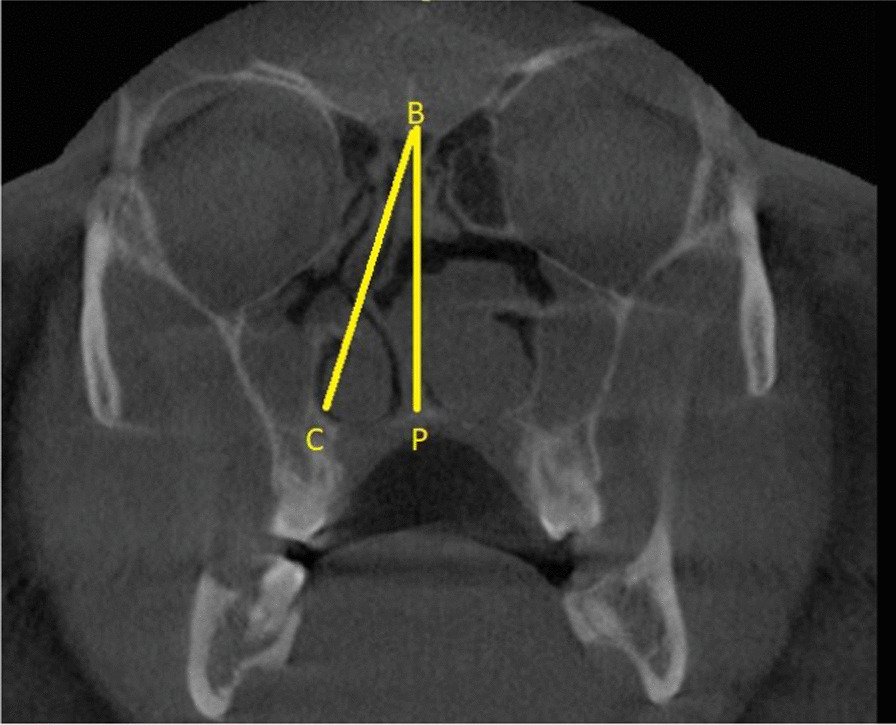
Coronal CBCT section showing the landmarks used for measuring the SDA. The thickening of the sinonasal mucosa was also observed in this image

The septal deviation angle (SDA) was measured in the coronal CBCT sections using the criteria of Kajan et al. and Orhan I et al. [10, 11]. The anatomical landmarks used for the measurement of the SDA are described in Fig. 1. Point P is the junction of the nasal septum with the floor of the nasal cavity. Point B is the Crista Galli. Line BC is the tangent arising from point B and passing through the outermost part on the convexity of the deviated septum. Angle PBC is the septal deviation angle (SDA).

**Fig. 2:**
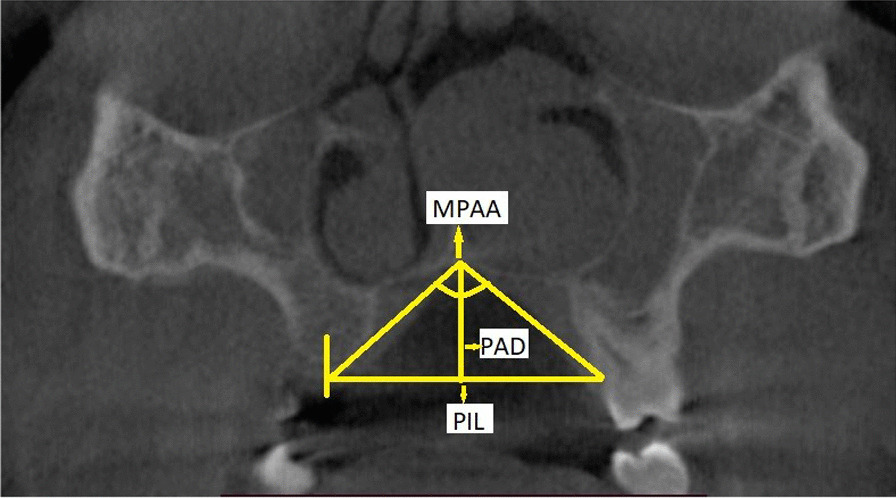
Coronal CBCT section showing landmarks for the palatal dimension measurements. The palatal interalveolar length (PIL) is the distance between the mid-centres of the cervical portion of the available tooth, from one side to the other. If there was no tooth, then the mid-centre of the alveolar bone near the crest was considered the reference point. Palatal arch depth (PAD) is the length of the line from "P" (junction of the nasal septum and hard palate) to the interalveolar line. The maxillopalatal arch angle (MPAA) is the angle that is formed by the lines from "P" to both points of the mid-centre of the available tooth or the midpoint maxillary alveolar bone for patients missing teeth

Palatal interalveolar length (PIL), palatal arch depth (PAD) and maxillopalatal arch angle (MPAA) were measured based on the criteria of Kajan et al. [9]. The radiographic landmarks used for the determination of PIL, PAD and MPAA are described in Fig. 2.

**Fig. 3:**
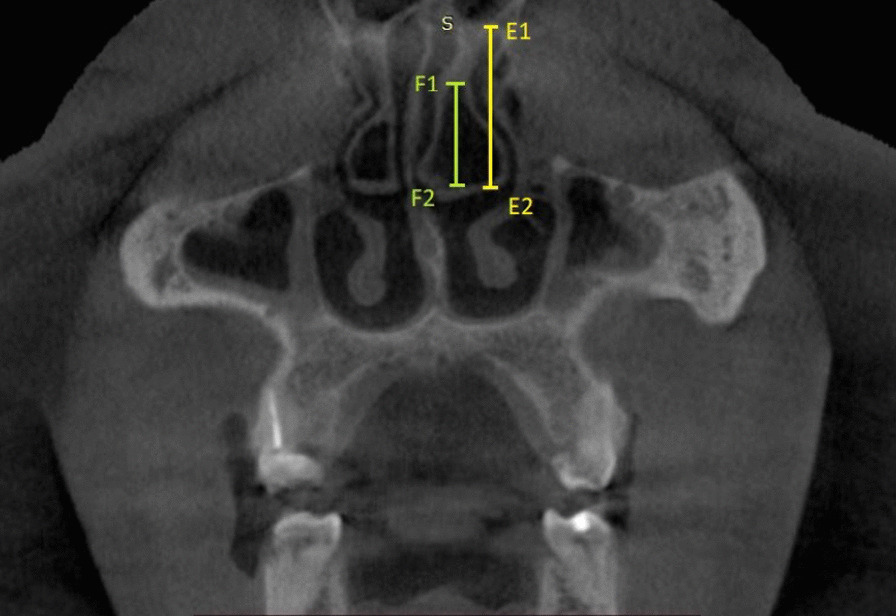
Coronal CBCT view showing the method used for identifying CB as per the criteria by Stallman et al. Line E1E2 represents the vertical length of the middle turbinate. Line F1F2 represents the extent of pneumatization caused by CB

The presence of CB was determined based on the criteria stated by Stallman et al. [6]. The presence of CB was confirmed only when more than 50% pneumatization of the vertical height of the middle turbinate was observed on the CBCT image (Fig. 3).

In Group 2 and Group 4, CBCT scans of subjects with anteroposterior C-shaped septal deviation were included. The data obtained after evaluation of the CBCT scans were statistically analysed using IBM SPSS statistics (Version 22, Armonk. NY: IBM Corp).

## Results

The intrarater reliability values for the two examiners were 0.84 and 0.86, respectively (Cohen kappa test). The interrater agreement between the two examiners was 0.81. Among the 200 study subjects, 63% (n = 126) were men and 37% (n = 74) were women. There was no significant difference (*p* = 0.65) in the ratio of male to female subjects among the 4 study groups (Table 1).

**Table 1:** Comparison of the gender distribution of the subjects in the study groups

Group				Total	Chi-square test	
1	2	3	4		Chi square value	*p* value
*Gender*						
Male						
60	35	18	13	126	1.67	0.65 (NS)
66.66%	63.63%	56.25%	56.52%	63%		
Female						
30	20	14	10	74		
33.33%	36.37%	43.75%	43.48%	37%		

Overall and post hoc comparisons of the mean age of the study subjects in the groups revealed no significant differences (*p* = 0.65) (Tables 2 and 3). When an overall comparison of the SDA, PIL, PD, and MPAA was made among the study groups, there was a statistically significant difference (*p* < 0.001). The PIL and MPAA were significantly lower in Groups 2, 3, and 4 than in Group 1. The PD was higher in Groups 2, 3, and 4 than in Group 1 (Table 2).

**Table 2:** Showing the overall comparison of the age, SDA, PIL, PD, and MPAA among the four study groups

Study groups	N	Mean	SD	Min	Max	ANOVA	
						F	*p* value
*Age*							
1	90	51.88	14.58	27	80	3.42	0.65 (NS)
2	55	49.98	14.65	20	70		
3	32	49.17	15.63	18	72		
4	23	51.4	14.82	27	80		
*SDA*							
1	90	0	0	0	0	226.27	< 0.001*
2	55	7.6	3.41	2	14		
3	32	0	0	0	0		
4	23	12.86	4.73	3	21		
*Palatal intralveolar length (PIL)*							
1	90	44.21	4.23	37.33	55.15	79.00	< 0.001*
2	55	39.81	1.93	36.06	44.15		
3	32	40.37	1.92	36.16	44.09		
4	23	35.91	1.87	33.03	40.22		
*Palatal depth (PD)*							
1	90	13.16	1.18	10.55	15.35	51.12	< 0.001*
2	55	14.90	1.58	10.48	16.93		
3	32	15.47	2.48	6.84	21.55		
4	23	17.35	1.26	14.02	20.45		
*Mid palatal arch angle (MPAA)*							
1	90	123.67	3.62	117.50	131.20	48.18	< 0.001*
2	55	119.73	2.26	116.00	124.00		
3	32	120.62	2.39	116.00	126.00		
4	23	116.81	2.94	110.00	123.10		

*p < 0.05 statistically significant, *p* > 0.05 Non significant, NS

**Table 3:** Post hoc tests for intergroup comparison age, SDA, PIL, PD, and MPAA among the four study groups

Dependent variable	(I) Group	(J) Group	Mean difference (I–J)	Std. error	*p* value	95% CI	
						Lower bound	Upper bound
Age	1	2	2.90	2.94	1.00(NS)	− 4.29	10.51
		3	2.71	2.94	0.79(NS)	− 4.90	10.32
		4	2.48	2.91	1.00(NS)	− 7.06	8.01
	2	3	2.19	2.95	0.85(NS)	− 7.84	7.54
		4	2.42	2.92	1.00(NS)	− 6.00	8.85
	3	4	2.23	2.92	0.87(NS)	− 9.81	5.34
SDA	1	2	− 7.60	0.60	< 0.001*	− 9.15	− 6.06
		3	0	0.595	1.00(NS)	− 1.54	1.54
		4	− 12.86	0.59	< 0.001*	− 14.39	− 11.33
	2	3	7.60	0.60	< 0.001*	6.05	9.16
		4	− 5.26	0.59	< 0.001*	− 6.79	− 3.72
	3	4	− 12.86	0.59	< 0.001*	− 14.40	− 11.32
Palatal intralveolar length	1	2	4.40	0.55	< 0.001*	2.98	5.81
		3	3.84	0.55	< 0.001*	2.43	5.25
		4	8.29	0.54	< 0.001*	6.89	9.69
	2	3	− 0.56	0.55	0.74(NS)	− 1.98	0.86
		4	3.90	0.54	< 0.001*	2.49	5.30
	3	4	4.45	0.54	< 0.001*	3.05	5.86
Palatal depth	1	2	− 1.75	0.35	< 0.001*	− 2.64	− 0.85
		3	− 2.31	0.35	< 0.001*	− 3.21	− 1.42
		4	− 4.19	0.34	< 0.001*	− 5.08	− 3.31
	2	3	− 0.57	0.35	0.37(NS)	− 1.46	0.33
		4	− 2.45	0.34	< 0.001*	− 3.34	− 1.56
	3	4	− 1.88	0.34	< 0.001*	− 2.77	− 0.99
Mid palatal arch angle	1	2	3.94	0.58	< 0.001*	2.43	5.44
		3	3.05	0.58	< 0.001*	1.54	4.55
		4	6.85	0.57	< 0.001*	5.36	8.34
	2	3	− 0.89	0.58	0.42(NS)	− 2.40	0.62
		4	2.92	0.58	< 0.001*	1.42	4.41
	3	4	3.81	0.58	< 0.001*	2.31	5.30

Tukey post hoc test

*p < 0.05 statistically significant, *p* > 0.05 Non significant, NS

Post hoc comparison revealed that there was a significant difference (*p* < 0.01) in the mean PIL, PD, and MPAA values among the groups. However, there was no significant difference (*p* = 1.00) in the mean SDA values between Group 1 and Group 3. Similarly, there was no significant difference in the mean PIL (*p* = 0.74), mean PD (*p* = 0.37), or mean PAL (*p* = 0.42) values between Group 2 and Group 3 (Table 3).

When the mean SDA, PIL, PD, and MPAA in study subjects with unilateral CB, bilateral CB and absence of CB were compared using ANOVA, there was a significant difference (*p* < 0.001) (Table 4). However, post hoc analysis revealed that there was no significant difference in the mean PIL (*p* = 0.86), mean PD (*p* = 0.32), or mean PAA (*p* = 0.61) between unilateral and bilateral CB (Table 5).

**Table 4:** Overall comparison of the PIL, PD, and PAA in study subjects with unilateral CB, bilateral CB and absence of CB

CB	N	Mean	SD	Min	Max	ANOVA	
						F	*p* value
*PIL*							
Absent	145	42.03	3.96	36.06	55.15	31.27	< 0.001*
Unilateral	40	38.17	2.82	33.06	44.09		
Bilateral	15	37.68	3.50	33.03	42.63		
*PD*							
Absent	145	14.02	1.64	10.48	16.93	39.50	< 0.001*
Unilateral	40	16.31	2.27	6.84	21.55		
Bilateral	15	17.06	1.39	14.25	19.44		
*MPAA*							
Absent	145	121.72	3.60	116.00	131.20	19.41	< 0.001*
Unilateral	40	118.83	3.21	110.00	126.00		
Bilateral	15	117.93	3.66	111.30	123.00		

*p < 0.05 statistically significant, *p* > 0.05 Non significant, NS

**Table 5:** Post hoc analysis of the SDA, PIL, PD and PAA in study subjects with unilateral CB, bilateral CB and absence of CB

Dependent variable	(I) CB	(J) CB	Mean difference (I–J)	Std. error	*p* value	95% CI	
						Lower bound	Upper bound
PIL	Absent	Unilateral	3.86	0.52	< 0.001*	2.62	5.09
		Bilateral	4.35	0.94	< 0.001*	2.13	6.57
	Unilateral	Bilateral	0.49	0.95	0.86(NS)	− 1.76	2.74
PD	Absent	Unilateral	− 2.28	0.29	< 0.001*	− 2.96	− 1.61
		Bilateral	− 3.04	0.52	< 0.001*	− 4.26	− 1.82
	Unilateral	Bilateral	− 0.75	0.52	0.32(NS)	− 1.99	0.48
MPAA	Absent	Unilateral	2.89	0.52	< 0.001*	1.67	4.11
		Bilateral	3.79	0.93	< 0.001*	1.60	5.99
	Unilateral	Bilateral	0.90	0.94	0.61(NS)	− 1.33	3.13

Tukey post hoc test

*p < 0.05 statistically significant; p > 0.05 Non significant, NS

When the mean SDA, PIL, PD, and PAA in study subjects with unilateral turbinate hypertrophy (TH), bilateral TH and absence of TH were compared using ANOVA, there was a significant difference (*p* < 0.001) (Table 6). However, post hoc analysis revealed that there was no significant difference in the mean PIL (*p* = 0.85), mean PD (*p* = 0.84), or mean PAA (*p* = 0.95) between unilateral and bilateral TH (Table 7). There was no significant difference between the mean PAA (*p* = 0.60) in subjects with an absence of TH and bilateral TH.

**Table 6:** Overall comparison of the PIL, PD, and MPAA in study subjects with unilateral TH, bilateral TH and absence of TH

Turbinate hypertrophy	N	Mean	SD	Min	Max	ANOVA	
						F	*p* value
*PIL*							
Absent	127	41.69	4.28	33.45	55.15	12.94	< 0.001*
Unilateral	60	38.96	3.34	33.03	49.04		
Bilateral	15	38.16	3.75	34.02	42.63		
*PD*							
Absent	127	14.24	1.86	10.55	18.76	15.10	< 0.001*
Unilateral	60	15.89	2.27	6.84	21.55		
Bilateral	15	16.36	2.52	12.50	19.44		
*MPAA*							
Absent	127	121.25	4.00	110.00	131.20	5.73	0.004*
Unilateral	60	119.43	3.48	110.00	129.30		
Bilateral	15	119.86	2.31	116.00	123.00		

*p < 0.05 statistically significant, *p* > 0.05 Non significant, NS

**Table 7:** Post hoc analysis of the SDA, PIL, PD, and MPAA in study subjects with unilateral TH, bilateral TH and absence of TH

Dependent variable	(I) Turbinate hypertrophy	(J) Turbinate hypertrophy	Mean difference (I–J)	Std. error	*p* value	95% CI	
						Lower bound	Upper bound
PIL	Absent	Unilateral	2.72	0.56	< 0.001*	1.41	4.04
		Bilateral	3.52	1.48	0.04*	0.02	7.03
	Unilateral	Bilateral	0.80	1.47	0.85(NS)	− 2.67	4.27
PD	Absent	Unilateral	− 1.66	0.31	< 0.001*	− 2.39	− 0.92
		Bilateral	− 2.12	0.83	0.03*	− 4.09	− 0.15
	Unilateral	Bilateral	− 0.47	0.83	0.84(NS)	− 2.42	1.48
MPAA	Absent	Unilateral	1.83	0.54	0.003*	0.55	3.11
		Bilateral	1.40	1.45	0.60(NS)	− 2.02	4.82
	Unilateral	Bilateral	− 0.43	1.43	0.95(NS)	− 3.82	2.95

Tukey post hoc test

*p < 0.05 statistically significant, *p* > 0.05 Non significant, NS

Multiple linear regression revealed a statistically significant association between SDA, CB and TH and PIL, PD, and MPAA (Table 8). However, the age and sex of the study subjects did not show a significant association with PIL, PD, or MPAA. PIL shows the highest association (R^2^ = 0.53) with SDA, CB and TH.

**Table 8:** Multiple linear regression to predict PIL, PD, and MPAA based on the study variables

Model		Unstandardized coefficients		Standardized coefficients	t	*p* value	95.0% CI for B	
		B	Std. error	Beta			Lower bound	Upper bound
PIL	(Constant)	43.24	0.92		46.85	< 0.001*	41.42	45.06
	Age	0.01	0.01	0.04	0.79	0.43 (NS)	− 0.02	0.04
	Gender	− 0.49	0.43	− 0.06	− 1.15	0.25 (NS)	− 1.34	0.35
	SDA	− 0.37	0.03	− 0.58	− 10.98	< 0.001*	− 0.44	− 0.31
	CB	− 2.30	0.39	− 0.37	− 5.90	< 0.001*	− 3.07	− 1.53
	TH	0.38	0.46	0.05	0.83	0.04*	− 2.52	− 1.28
PD	(Constant)	14.53	0.60		24.42	< 0.001*	13.36	15.71
	Age	− 0.02	0.01	− 0.10	− 1.78	0.08 (NS)	− 0.03	0.00
	Gender	− 0.10	0.28	− 0.02	− 0.35	0.73 (NS)	− 0.64	0.45
	SDA	0.12	0.02	0.34	5.68	< 0.001*	0.08	0.17
	CB	1.65	0.25	0.47	6.56	< 0.001*	1.15	2.15
	TH	0.08	0.30	0.12	0.28	0.03*	1.67	2.50
MPAA	(Constant)	122.80	0.96		128.35	< 0.001*	120.91	124.68
	Age	0.01	0.01	0.03	0.50	0.62 (NS)	− 0.02	0.03
	Gender	− 0.49	0.45	− 0.06	− 1.09	0.28 (NS)	− 1.36	0.39
	SDA	− 0.33	0.04	− 0.55	− 9.44	< 0.001*	− 0.40	− 0.26
	CB	− 2.19	0.41	− 0.37	− 5.41	< 0.001*	− 2.99	− 1.39
	TH	1.12	0.47	0.16	2.35	0.02*	0.18	2.05

Mid palatal intralveolar length—F(5, 189) = 42.87, *p* < 0.001, R^2^ = 0.53. Palatal depth F(5, 189) = 24.75, *p* < 0.001, R^2^ = 0.40. Palatal arch angle F(5, 189) = 28.87, *p* < 0.001, R^2^ = 0.43.

*p < 0.05 statistically significant, *p* > 0.05 Non significant, NS

There was no significant correlation between PIL, PD, and MPAA and SDA in Group 2. However, in Group 4, SDA showed a significant correlation with PIL (*p* < 0.001), PD (*p* = 0.67) and MPAA (*p* = 0.02) (Table 9).

**Table 9:** Correlation between PIL, PD, MPAA and SDA

		PIL	PD	MPAA
Group 2	r	− 0.28	0.02	− 0.21
	*p* value	0.051 (NS)	0.92 (NS)	0.15 (NS)
Group 4	r	− 0.66	0.67	− 0.63
	*p* value	< 0.001*	0.01*	0.02*

Pearsons’ correlation test

*p < 0.05 statistically significant, *p* > 0.05 Non significant, NS

## Discussion

The growth of the nasal palatine complex and paranasal sinuses is believed to be influenced by factors such as nasal airflow, brain development and orofacial musculature strength [12–15]. Recent studies have found that the presence of CB and TH are associated with the occurrence of NSD [11, 16]. With this background, we conducted a study to evaluate changes in palatal dimensions associated with the occurrence of NSD, CB and TH.

In the present study, there was no significant difference in the age or gender distribution among the study groups. However, there was a significant difference in the SDA, PIL, PD and MPAA among the four study groups. The SDA was significantly higher in Group 4 than in Group 2, indicating that the presence of CB was associated with a higher degree of septal deviation. Similar results were obtained in the studies by Yigit et al. and El−Taher et al. [17, 18]. The exact cause-effect relationship for this association is still unclear [19]. One theory states that the presence of NSD facilitates further pneumatization of a pre-existing CB, depending on the degree of SDA [5]. The other theory states that NSD may be caused by CB [20].

In the present study, PIL and MPAA were significantly higher in Group 1 than in Groups 2, 3, and 4. The PD was significantly lower in Group 1 than in the other groups. Similar findings were reported by Kajan et al., Sapmaz et al., Akbay et al. and Ascanio et al. [10, 12, 13, 15]. In the study by Akbay et al., computed tomography (CT) was used to analyse the association between NSD and the palatal dimensions [13]. Ascanio et al. used cephalometrics in their study to determine NSD and the palatal dimensions [15]. Sapmaz et al. used CT scans in their study to determine the association between NSD, maxillary sinus volume and the angle of the hard palate [12]. The effect of CB and TH was not considered in the studies by Sapmaz et al., Akbay et al. and Ascanio et al. [12, 13, 15]. In a study by Kajan et al., the presence of CB was considered while analysing the association of NSD with palatal dimensions [10]. However, in the study by Kajan et al., the decrease in the PIL and MPAA in subjects with NSD and CB was not statistically significant. In the present study, the difference was significant. This difference could be attributed to the larger sample size in our study compared to the study by Kajan et al. The probable cause for the alterations in palatal dimensions in the presence of CB could be attributed to nasal obstruction [21]. It is believed that oral respiration resulting from nasal obstruction causes an increase in palatal depth. This increased palatal depth over time further stimulates the existing deviation [22].

In the present study, there was no significant difference in the PIL, PD or MPAA between subjects with unilateral CB and bilateral CB. When CB occurs bilaterally, one side is often dominant or larger than the other. Septal deviation occurs on the opposite side of the dominant CB [23]. This could be a reason for the absence of a significant difference in the palatal dimensions in unilateral and bilateral CB.

In the present study, there was a significant difference in the palatal dimensions of the study subjects with TH compared to subjects without TH. TH is one of the common causes of nasal airway obstruction [24]. Nasal airway obstruction is associated with a reduction in the intra-alveolar width and an increase in palatal depth [25].

In the present study, SDA, CB and TH had significant effects on the PIL. Earlier studies have demonstrated that the presence of nasal obstruction and septal deviation are associated with decreased palatal width [26]. However, there are very few studies that have evaluated the association between CB and TH on palatal width. Researchers have found that uninterrupted airflow through the air passage induces a persistent stimulus for the horizontal growth of the maxilla and for lowering the palatal vault and increasing the palatal intra-alveolar width [27].

In the present study, there was a significant positive correlation between SDA and PD. Similar results were obtained by Akbay et al., who observed a strong positive correlation between septal deviation and the depth of the maxillopalatal arch [13]. A similar finding was observed in a study by Wang et al. in a CT-based study [28]. However, in a study by Serter et al., the palatal depth was decreased in subjects with nasal polyps [29], but no clear reason was provided for the finding in this study. In the present study, there was a significant negative correlation between SDA and MPAA. In a study by Sapmaz et al., there was a positive correlation between SDA and the angulation of the hard palate. The difference in the results could be due to the method of determining the angulation. In the study by Sapmaz et al., the angulation of the hard palate was calculated using a reference line drawn parallel to the line joining the lesser wings of the sphenoid in the coronal CT section [12].

The results from the study by Sapmaz et al. suggest that alterations in the angulation of the hard palate are more likely to be caused by nasal septum deviation rather than a reduced volume of the maxillary sinus. This is contrary to the findings of research studies associating paranasal sinus volumes with nasal obstruction [30]. In the study by Sapmaz et al., the angulation of the hard palate caused no significant differences in the maxillary sinus volumes.

In the present study, the PIL, PD and MPAA had significant correlations with SDA in Group 4 (NSD + CB), whereas the correlation was not significant in Group 2 (NSD only). This could be explained by the results of the study conducted by Awuapara et al. [31]. In their study, they found no significant correlation between SDA and the palatal dimension. Most likely, the presence of CB in the study subjects in Group 4 was the reason for the significant correlation in the present study.

The clinical implications of the results from the present study indicate that nasal airway passage blockade caused by NSD with CB could impact the growth and downwards arching of the palate. Therefore, early intervention and management of NSD and CB are important to avoid the progression of maxillopalatal deformity and malocclusion. The data from the present study could be useful in evaluating the nasal and craniofacial alterations occurring during and after rapid maxillary expansion, particularly in adolescent patients. Recent studies have evaluated the skeletal changes occurring in the nasal cavity, craniofacial region and even the mandible after rapid maxillary expansion [32, 33].

### Limitations and future scope

One of the major limitations of this study is that all the parameters were measured in two-dimensional aspects. Recent studies have revealed that segmentation and three-dimensional volumetric analyses can be very useful in evaluating oronasal structures [34]. The volumetric analysis of soft tissue structures such as nasal turbinates and air-filled structures like the conch bullosa can be performed using CBCT scans and advanced software [34]. A recent study using a deep learning-based method for automated segmentation of the sinonasal region in CBCT scans revealed that the volumetric measurements were as accurate as those performed by an experienced maxillofacial radiologist [35].

## Conclusion

From the outcomes of the present study, we can conclude that NSD and CB can cause significant alterations in palatal dimensions. There was no significant change in the palatal dimensions between unilateral and bilateral CB. Among the palatal dimensions, PIL has the most significant association with SDA and CB. There was a significant correlation between the palatal dimensions and SDA when CB was present along with NSD. Therefore, we can conclude that variations in the nasal cavity, such as NSD, CB and TH, can contribute to palatal dimensions and skeletal malocclusion. However, prospective studies with larger sample sizes are necessary to validate the findings from our study.

## Data Availability

The corresponding can be contacted for raw data. The data is also available at figshare; https://doi.org/10.6084/m9.figshare.14852373.
